# Commentary: Bibliometric analysis of surface electromyography trends in stroke rehabilitation research

**DOI:** 10.3389/fneur.2026.1771446

**Published:** 2026-02-02

**Authors:** Hong Liu, Mi Huang, Zaixiang Zhang, Hua Zhao

**Affiliations:** Hubei University of Chinese Medicine Affiliated Gong'an Hospital of Traditional Chinese Medicine, Jingzhou, China

**Keywords:** bibliometric analysis, clinical application, rehabilitation, sEMG, stroke

## 1 Introduction

Stroke ranks among the most prevalent diseases globally, posing a significant threat to life and health. Despite variations in healthcare systems across nations, addressing the burden of stroke remains a global priority (1). Bibliometric analysis plays an increasingly vital role in medical research, primarily due to its ability to quantitatively and qualitatively assess research trends and hotspots within specific disciplines (2). This method efficiently analyzes the quantitative characteristics and patterns of literature in stroke research, providing directional guidance and reference for future studies. However, greater emphasis should be placed on methodological rigor and scientific validity when conducting bibliometric research.

## 2 Commentary

We read with great interest the publication edited by Liao et al. (3) titled “Bibliometric analysis of surface electromyography trends in stroke rehabilitation research,” which was published in the issue of Frontiers in Neurology. By using bibliometrics, this study has identified the countries, top institutions, journals, and researchers globally involved in sEMG research on stroke. Keyword analysis shows that “rehabilitation” and “recovery” are key research themes, reflecting a focus on improving post-stroke functional outcomes. The analysis reveals that future research should focus on the clinical utility of sEMG, including cost-effectiveness and integration into clinical workflows, while developing clear application guidelines. We highly support and appreciate the researchers' studies, and we thank them for their contributions to the field. However, we identified several points requiring clarification and revision.

**Figure 1:**
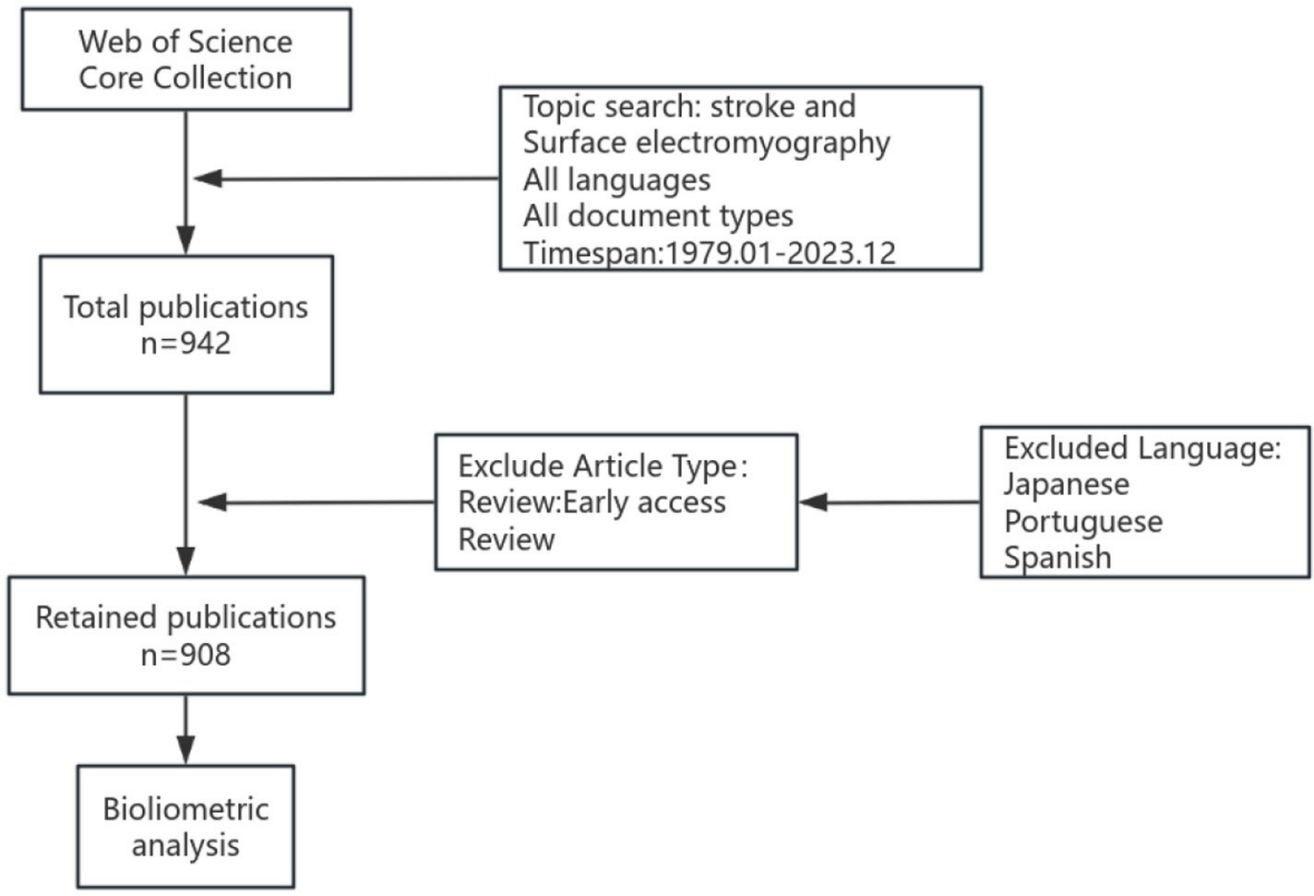
The flowchart of papers identification and selection.

First, as Figure 1 showed, the study used non-standard inclusion criteria in its bibliometric analysis, incorporating diverse document types such as editorials, letters, conference abstracts, and conference papers while excluding reviews and early access reviews. Reviews are crucial for mapping a field's academic landscape and emerging trends, providing a more accurate reflection of significant directions and research hotspots within a specific domain. Editorials and conference abstracts typically represent preliminary or non-original research. This selection may skew the dataset, potentially inflating publication counts for certain institutions or authors without corresponding contributions to substantive research environments. We strongly question this methodological decision, and the original authors should provide explicit justification. We recommend the authors rerun key analyses (e.g., top institutions, prolific authors, and keywords) using a more traditional dataset (excluding document types other than “articles” and “reviews”) to demonstrate the robustness—or lack thereof—of their primary conclusions. Bibliometric studies, such as Zhang et al., provide valuable comparative benchmarks for sound methodology and deeper thematic analysis (4).

**Figure 2:**
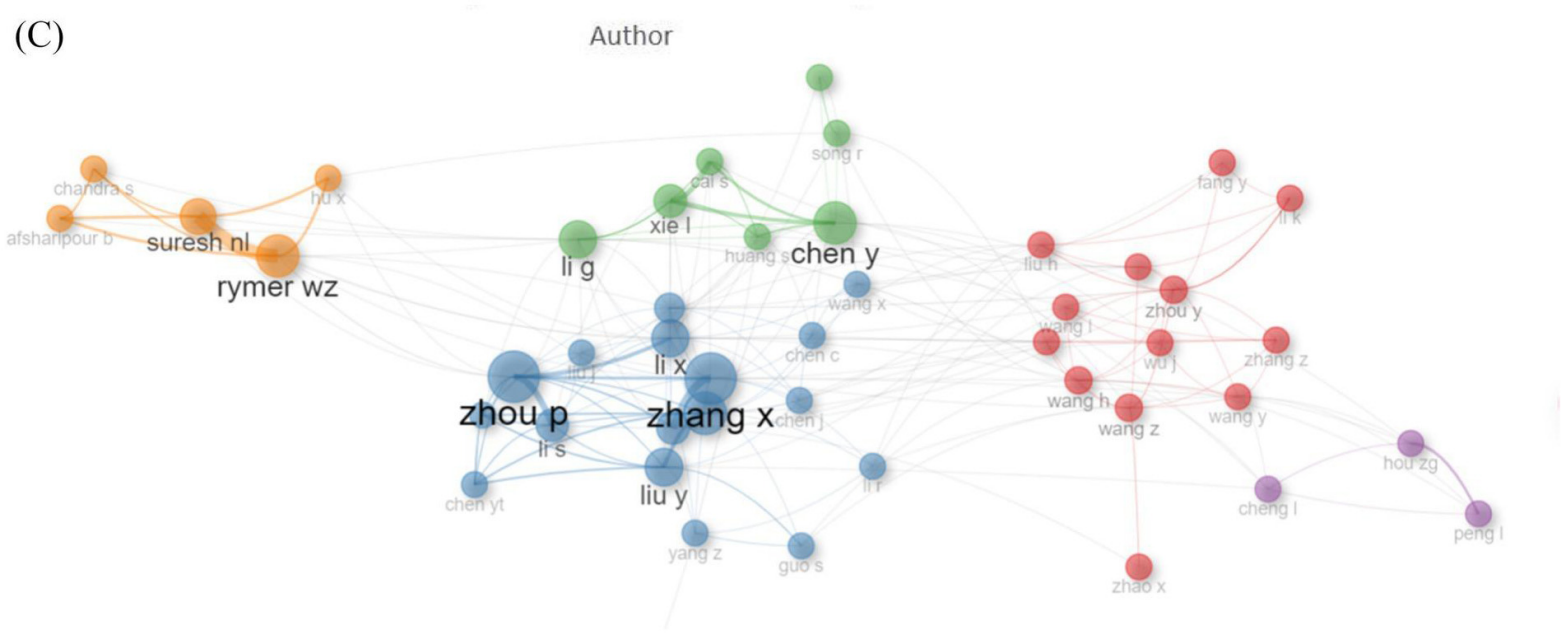
Authors' Local Impact by H-index. H-index is an index used to measure the academic influence of scholars, scientists or researchers in bibliometrics. Specifically, if a scholar's H-index is 10, it means that the scholar has at least 10 papers, each of which has been cited at least 10 times by other scholars.

Second, the original study presents contradictory narratives regarding author influence. In the text, Lay B.S. is rated as the “most influential” author based on 120 total citations. However, Figure 2 in the same section visually represents author influence using the H-index, where Zhang X and Zhou P lead with an H-index of 11. The absence of a unifying framework in these statements, which are based on disparate metrics, results in a state of perplexity. This represents a substantial deficiency in the processes of data interpretation and reporting. Additionally, the study's evaluation of author influence is insufficient and incomplete, relying solely on a limited set of metrics (publication volume and H-index). We advocate that robust bibliometric assessments of author impact require transparent, multi-metric approaches (e.g., total citations, average citation count, citations per paper, H-index, and G-index) rather than selective reporting. The original text's assertion about “most influential authors” currently lacks substantiation and necessitates correction or a more nuanced explanation.

Third, the original study's keyword analysis was limited to listing high-frequency terms such as “stroke” and “rehabilitation.” This approach fails to reveal the field's dynamic knowledge structure beyond a superficial snapshot. Specifically, we recommend that the original authors conduct keyword co-occurrence network analysis and clustering to map distinct research sub-themes (e.g., “robot-assisted therapy,” “brain-computer interfaces,” and “muscle synergies”). Furthermore, analyzing thematic evolution across different time periods is crucial for visualizing shifts in research focus (e.g., from basic assessment to technology integration). Such deeper analysis transforms simple counts into meaningful insights about past trends and future directions.

The research indicates that future studies should focus on clinical utility, however its discussion fails to use bibliometric findings to critically analyze specific barriers to clinical application. It is recommended that the authors synthesize their results, including the prominence of engineering-focused, highly cited papers vs. patient-centered outcome research, to structure a discussion on translational challenges. These may include the lack of standardized sEMG protocols, cost-effectiveness studies, clinical practice guidelines, and interoperability with routine clinical workflows.

In summary, the authors' contributions to the study of surface electromyography trends in stroke rehabilitation are acknowledged. It is further recommended that more rigorous research methodologies and evaluation metrics be adopted to enhance the study's scientific rigor.
